# Assessment of deep convolutional neural network models for species identification of forensically-important fly maggots based on images of posterior spiracles

**DOI:** 10.1038/s41598-022-08823-8

**Published:** 2022-03-19

**Authors:** Darlin Apasrawirote, Pharinya Boonchai, Paisarn Muneesawang, Wannacha Nakhonkam, Nophawan Bunchu

**Affiliations:** 1 Department of Business Administration, Faculty of Business Economics and Communications, Naresuan University, Muang, Phitsanulok, 65000 Thailand; 2 Department of Electrical and Computer Engineering, Faculty of Engineering, Naresuan University, Muang, Phitsanulok, 65000 Thailand; 3 Department of Microbiology and Parasitology, Faculty of Medical Science, Naresuan University, Muang, Phitsanulok, 65000 Thailand

**Keywords:** Ecology, Zoology, Engineering

## Abstract

Forensic entomology is the branch of forensic science that is related to using arthropod specimens found in legal issues. Fly maggots are one of crucial pieces of evidence that can be used for estimating post-mortem intervals worldwide. However, the species-level identification of fly maggots is difficult, time consuming, and requires specialized taxonomic training. In this work, a novel method for the identification of different forensically-important fly species is proposed using convolutional neural networks (CNNs). The data used for the experiment were obtained from a digital camera connected to a compound microscope. We compared the performance of four widely used models that vary in complexity of architecture to evaluate tradeoffs in accuracy and speed for species classification including ResNet-101, Densenet161, Vgg19_bn, and AlexNet. In the validation step, all of the studied models provided 100% accuracy for identifying maggots of 4 species including *Chrysomya megacephala* (Diptera: Calliphoridae), *Chrysomya* (*Achoetandrus*) *rufifacies* (Diptera: Calliphoridae), *Lucilia cuprina* (Diptera: Calliphoridae), and *Musca domestica* (Diptera: Muscidae) based on images of posterior spiracles. However, AlexNet showed the fastest speed to process the identification model and presented a good balance between performance and speed. Therefore, the AlexNet model was selected for the testing step. The results of the confusion matrix of AlexNet showed that misclassification was found between *C. megacephala* and *C.* (*Achoetandrus*) *rufifacies* as well as between *C. megacephala* and *L. cuprina*. No misclassification was found for *M. domestica*. In addition, we created a web-application platform called thefly.ai to help users identify species of fly maggots in their own images using our classification model. The results from this study can be applied to identify further species by using other types of images. This model can also be used in the development of identification features in mobile applications. This study is a crucial step for integrating information from biology and AI-technology to develop a novel platform for use in forensic investigation.

## Introduction

The fly larvae of *Chrysomya megacephala* (Diptera: Calliphoridae), *Achoetandrus rufifacies* (Diptera: Calliphoridae), *Lucilia cuprina* (Diptera: Calliphoridae), and *Musca domestica* (Diptera: Muscidae) have long been recognized as crucial clues in medicolegal forensic entomology, especially in Thailand^1,2^. The estimation of the minimum postmortem interval (PMIm) from fly maggots plays a primary role in estimating the postmortem interval^3^. Moreover, human myiasis, the infestation of fly maggots in a living person, may be found in neglected people^1^. Therefore, fly larvae can be used to estimate the minimum abandonment duration^1^. The accuracy of PMIm determination and abandonment duration mainly relies on species identification^3^.

Identification of fly maggots is more difficult because the general morphology of each species is quite similar^4–6^. It is vermiform, circular in cross section, tapering to a point, and having no legs^4–6^. Morphology of the posterior spiracle is one of the important characteristics that have been used for identification of the larval stage^4–6^. Posterior spiracles are the organs involved in the gas exchanges, which are in the last segment and are joined to the dorsal longitudinal trunks of the internal tracheal system. The spiracles are placed in two chitinized plates surrounded by the peritreme. Typically, the number of slits can be used to classify the stage of larvae^6,7^. For example, the third-stage larva has three slits in each side of the posterior spiracle. The pain points of species identification based on morphological characteristics are the requirement of taxonomic experts and time consuming. In addition, the number of taxonomists and classification experts has drastically decreased so far. Although molecular techniques and proteomic analysis have been currently used for identification of fly species, specific equipment and reagents are required in the laboratory^8–10^.

To overcome the pain point, alternative automatic identification methods with expert-level classification accuracy are highly required in this field. Automatic species identification is important not only for flies in forensics, but also for other insects in general since it contributes to various purposes such as environment monitoring, pest diagnostics, and vector epidemiology^11^. Many previous studies indicated that automatic classification of insect species achieved high classification accuracy^12–18^. Currently, many classification models based on convolutional neural networks (CNNs) have been proposed in the field of computer vision. CNN is the type of multi-layered network learning algorithm, which usually contains multiple convolution layers, batch normalization layers, and fully connected layers^19^. Many previous reports demonstrated that the CNNs models have achieved species identification of insects with high accuracy and precision^12–18^. Based on our information, there have been no reports on the identification modeling of forensically-important fly maggots based on CNNs. Therefore, the novel method was proposed and the identification model was developed in 2 parts, including a custom object detection model for detection of position by using LabelImg, and YOLO programs, consecutively, and the multiple deep learning models to identify posterior spiracle images of four fly species including *C. megacephala* (Diptera: Calliphoridae), *C.* (*Achoetandrus*) *rufifacies* (Diptera: Calliphoridae), *L. cuprina* (Diptera: Calliphoridae), and *M. domestica* (Diptera: Muscidae). In this study, the assessment of the identification was compared with state-of-the-art CNNs including ResNet-101, Densenet161, Vgg19_bn, and AlexNet. The best performance model was selected and used to develop a web-application for automatic species identification.

## Materials and methods

### Preparation of posterior spiracle

Slides of the posterior spiracle were prepared following the method of Bunchu et al.^20^. Briefly, the morphology of the posterior spiracle was investigated by using the hydroxide clearing method. Maggots of each species (*C. megacephala*, *C*. (*A*.) *rufifacies*, *L. cuprina*, and *M. domestica*) were laboratory strains, which were obtained from the Medical Entomology Laboratory, Department of Microbiology and Parasitology, Faculty of Medical Science, Naresuan University. Adults of all species were identified by using the taxonomic key of Kurahashi and Bunchu^21^. The posterior spiracles of each species were photographed under the light microscope connected to the digital camera. All the images were used in the training process. For demonstration purposes, example images of each species were inverted using the rgb colors from rgb (255, 255, 255) to rgb (0, 0, 0) by using the image adjustment function of Adobe photoshop 2021 to increase the clarity of the images.

### Image data set

In this study, the images of the posterior spiracles of four forensically-important fly species as mentioned above were analyzed. All original images have been categorized as “verified” after they were identified and confirmed by an expert. In total, 17,144 original images were used in this study and divided into training (12,000 images), validation (3428), and testing (1716) groups with a number ratio of 7:2:1. For each species, the number of images for training, validation, and testing were 3000, 857, and 429 respectively.

**Figure 1:**
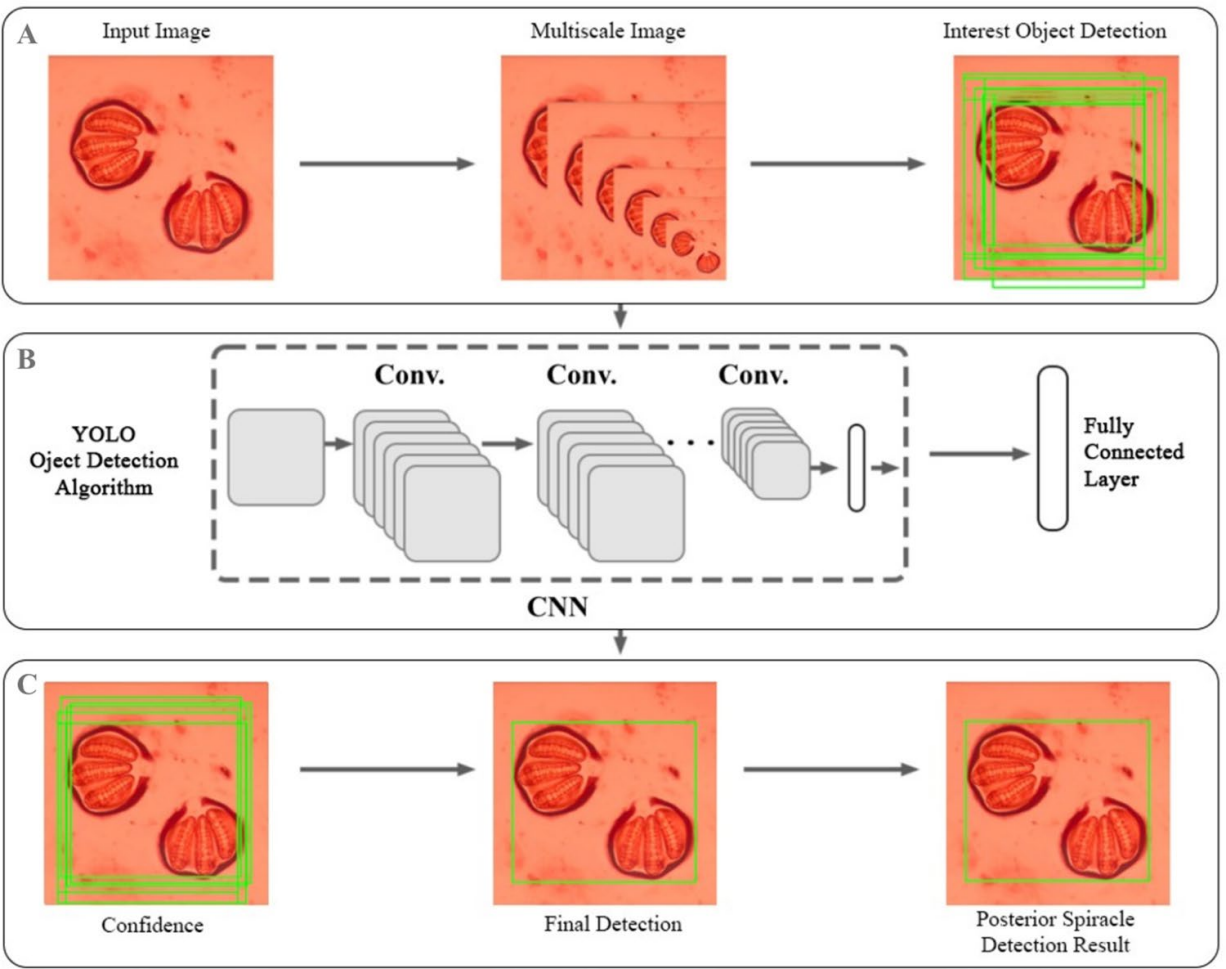
The processes of the custom object detection model used in this study was developed by using LabelImg program and YOLO object detection algorithm consisting 3 steps; (**A**) preparation process, (**B**) training process, and (**C**) detection process.

In the first step, we developed a custom object detection model for detecting the position of posterior spiracle in the input image by using LabelImg program and YOLO object detection algorithm. The process of the custom image detection was shown in Fig. 1. Briefly, this process divided the input image into subregions to predict multiple bounding boxes with their class probabilities for each region, training to create the most accurate object detection model for bounding boxes. Five hundred images were used for training the object detection model. The program used for labeling data for YOLO training was the LabelImg program. For the first step, the input images were added to the model to generate images in multiple scales. Then, the LabelImg program was used to label the position of the posterior spiracle in the image to detect the object of interest for further training (Fig. 1A). After that, the labeled images were used in training of the convolutional neural network (CNN) using YOLO object detection algorithm. The learning process was repeated to adjust the parameter several times to receive the highest accuracy. After passing through several convolutional layers, a fully connected layer was obtained to adjust the model output for detecting the position of the object in the image within bounding boxes (Fig. 1B). Therefore, this training model can be used to label the input image with the maximum confidence to select the final detection. The images were cropped tightly to the position of posterior spiracle (final detection). The purpose of this process was to ensure that each image was labeled precisely. This custom object detection data set was encoded to the JSON format with 224 × 224 pixels (detection result) (Fig. 1C). The cropped images with resolutions lower than 224 × 224 pixels were discarded from this study. The image data set was encoded to the JPEG format for the model training in the next step. This custom object detection model provided confidence and accuracy ≥ 80%.

### Model training

In this study, we compared the performance of four widely used models that vary in complexity of architecture to evaluate tradeoffs in accuracy and speed for species classification including ResNet-101, Densenet161, Vgg19_bn, and AlexNet. In the first step, we used the model from the previous step as mentioned above to speed up the training process. After randomly shuffling images in each species, the images within the species were divided by 7:2:1 ratio into training, validation, and testing groups. For each model, cropped images were resampled to the standard size for the input model. After using these models initially, we used 224 × 224 pixel images for Resnet-101, Densenet161, Vgg19_bn, and AlexNet. We used an image augmentation strategy following the previous study by Spiesman et al.^16^. Stochastic gradient descent (SGD) optimizer with an initial learning rate of 0.01 for all models was used. We used batch normalization, and models were trained for the 50 epochs using Nvidia K80, T4, P4 and P100 GPUs. At the species level, two metric model performances were calculated, including precision (1), recall (2), accuracy (3), and F1 score (4). Macro precision or mean of species-level precision scores and macro-recall or mean of species-level recall scores for each model were determined. The formulas for these indicators are as follows:

1 $$Precision=\frac{Tp}{Tp + Fp},$$

2 $$Recall=\frac{Tp}{Tp + Fn},$$

3 $$Accuracy=\frac{Tp + Tn}{Tp + Fp + Tn + Fn},$$

4 $$F1 score=\frac{2 {\times} Precision {\times} Recall}{Precision + Recall},$$

where *Tp, Tn, Fp,* and *Fn* represent the number of true positive, true negatives, false positive and false negatives, respectively. For the support value, it is the number of actual occurrences of the class in the specified dataset.

In addition, a confusion matrix table was constructed to realize a class-wise comparison to examine the accuracy of the identifications (generalized accuracy) and ensure that the model could detect and classify objects in a reliable manner. The mean accuracy determined from the table was analyzed and assessed for all the models. The mean time needed to predict the image in the test data set was also quantified as the model speed. All speed tests run on the same system using Nvidia K80s, T4s, P4s and P100s GPUs. The best performance model from this study was selected and used to develop web applications for automatic species identification.

To confirm the results of this study, we tested this model with other images from outsources (internet and personal contact) and visualized the results using the PyTorch CNN visualizations^22^.

## Results and discussion

**Figure 2:**
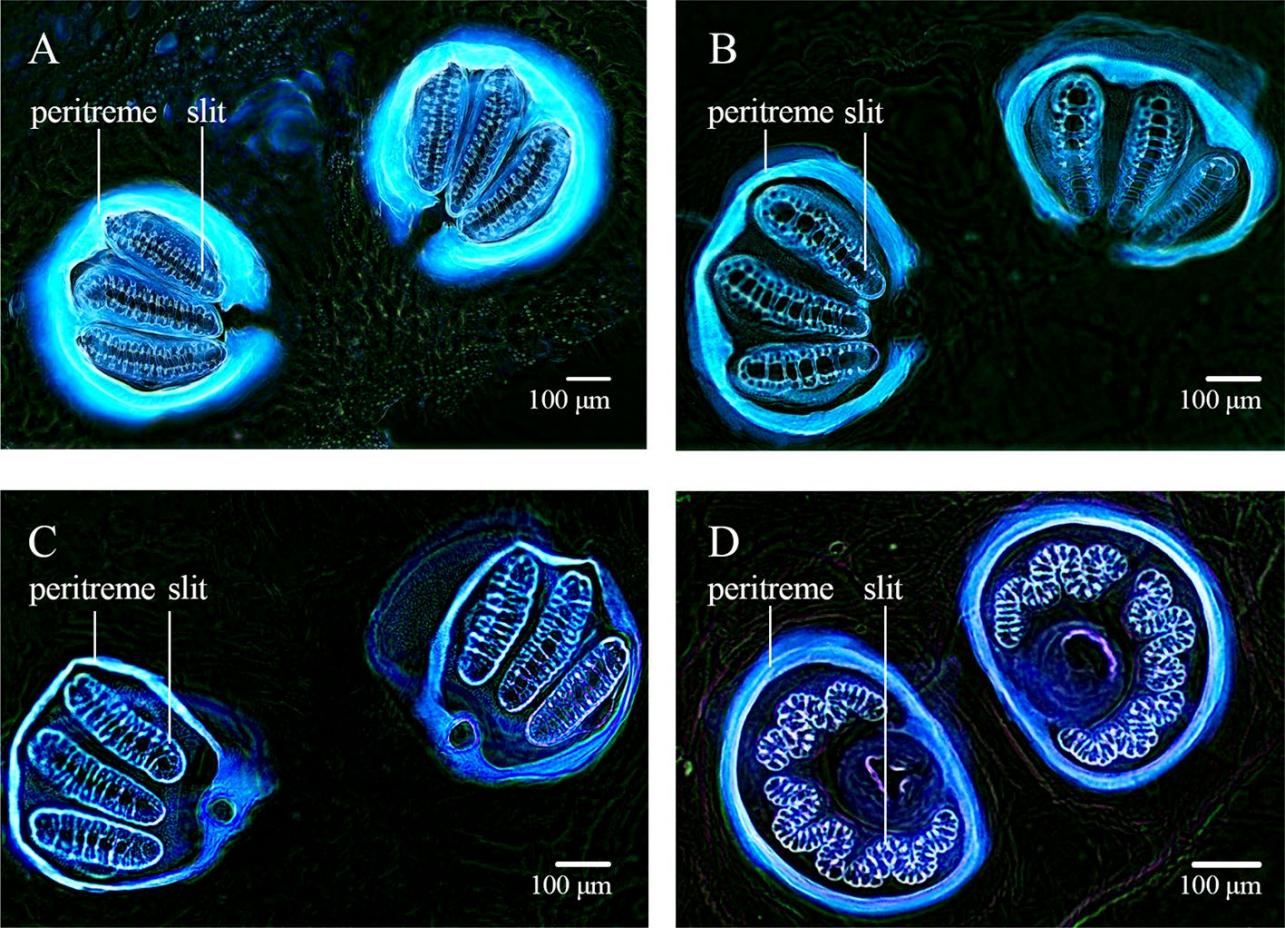
Morphology of posterior spiracles of four different fly species after inverting the image colors; (**A**) *Chrysomya* (*Achoetandrus*) *ruffifacies*, (**B**) *Chrysomya megacephala*, (**C**) *Lucilia cuprina*, (**D**) *Musca domestica*.

Of which at the third instar, the external morphology of larvae is quite similar; thus, the morphological identification used to differentiate between its genera or species, generally includes cephalophalyngeal skeleton, anterior spiracle, and posterior spiracles. The morphology of the posterior spiracle is one of the important characteristics for identification. A typical morphology of the posterior spiracle of third stage larvae was shown in Fig. 2. Based on studying under light microscopy, the posterior spiracle of *M. domestica* was clearly distinguished from the others. On the other hand, the morphology of the posterior spiracle of *C. megacephala* and *A. rufifacies* was quite similar. For *C. megacephala* and *C. rufifacies*, the peritreme, a structure encircling the three spiracular openings (slits), was incomplete and slits were straight as shown Fig. 2A,B, respectively. The complete peritreme encircling three slits was found in *L. cuprina* and *M. domestica* as shown in Fig. 2C,D, respectively. However, only the slits of *M. domestica* were sinuous like the M-letter (Fig. 2D). Their morphological characteristics found in this study were like the descriptions in the previous reports^23–25^.

For model training, four of the CNN models used for species-level identification of fly maggots provided 100% accuracy rates and 0% loss. Number of parameter (#Params), model speed, model size, macro precision, macro recall, f1-score, and support value were also presented in Table 1. The result demonstrated that the AlexNet model provided the best performance in all indicators when compared among four models. The AlexNet model used the least number of parameters while the Resnet101 model used the most. For model speed, the AlexNet model provided the fastest speed, while the Densenet161 model provided the slowest speed. For the model size, the AlexNet model was the smallest, while the Resnet101 model was the largest which corresponded to the number of parameters used. Macro precision, macro recall, f1-score and support value of all models were the same.

**Table 1:** Comparison of model size, speed, and performances of each studied model (The text in bold indicates the best value in each category).

Model	#Params (million)	Model speed (s)	Model size (mb)	Macro precision	Macro recall	f1-score	support
Resnet101	44.6	246.0	170.96	1.00	1.00	1.00	3388
Densenet161	14.2	326.4	55.33	1.00	1.00	1.00	3388
Vgg19_bn	20.6	210.0	78.67	1.00	1.00	1.00	3388
AlexNet	**2.7**	**10.8**	**10.58**	1.00	1.00	1.00	3388

As the training results presented in the supplementary data (Fig. S1), all models provided 100% accuracy and 0% loss in the early stage of training (< 10 epochs). This could be due to the training and testing processes with cropping specific portions of the fly images using our custom object detection model. Moreover, all images were from the laboratory strains, and their variation of morphological characteristics might be less than their wild counterparts. As a result, the training time was short, and the accuracy of the model was high. Of the four models tested, AlexNet demonstrated a good balance between performance and speed. This model can proceed the system the fastest and its model size was the smallest. The speed and accuracy of AlexNet made it useful for web-based and mobile applications that relied on both speed and reliable predictions. Speed was a factor in user satisfaction and would be important for future development such as video-based applications^16^. Therefore, we focused on the AlexNet results for the remainder of this article.

**Figure 3:**
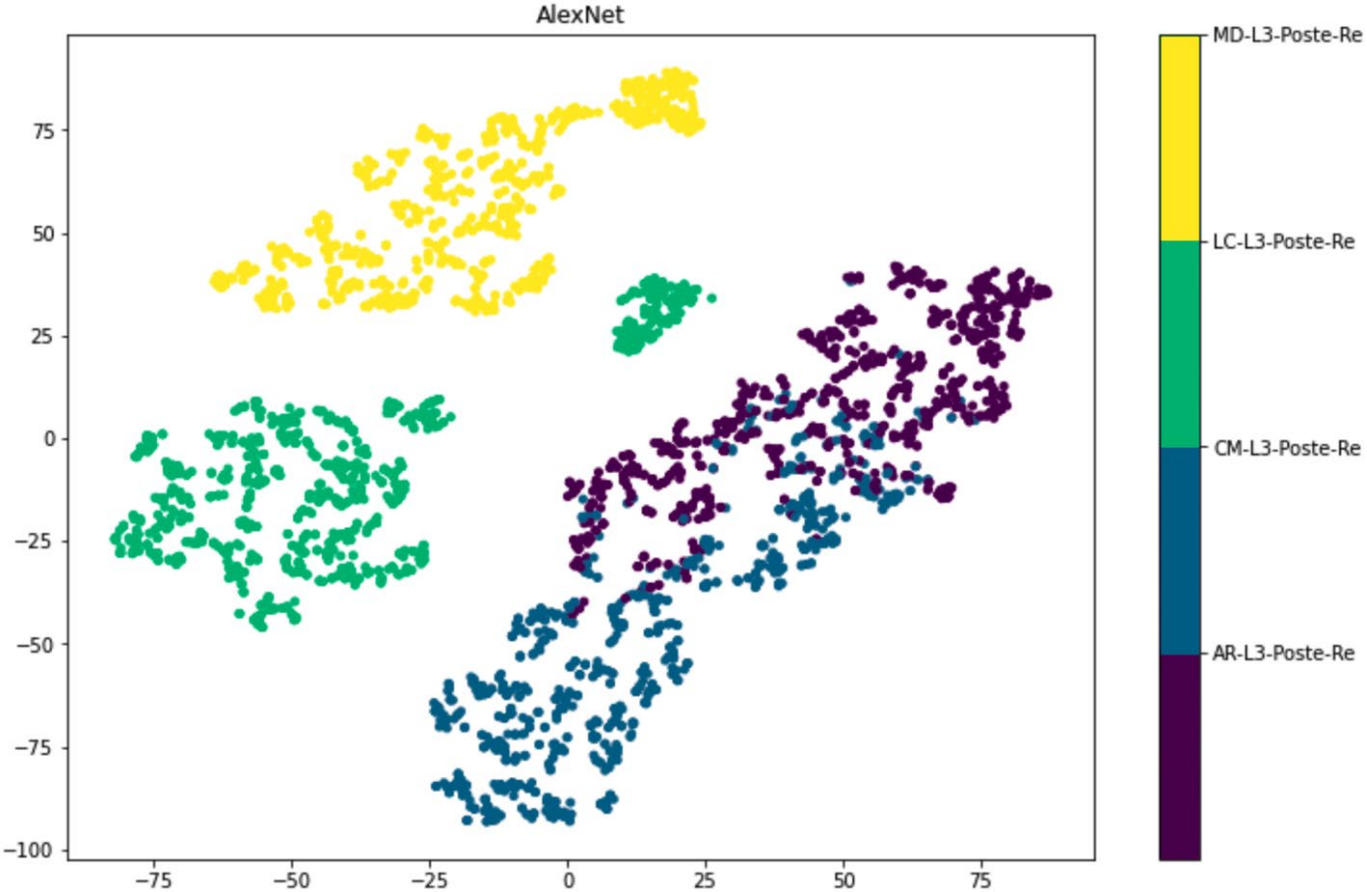
tSNE visualization of the AlexNet model by dimensionality reduction of the penultimate features (The test data are shown in colors for different classes; yellow refers to data set of *Musca domestica*, green refers to data set of *Lucilia cuprina*, greenish blue refers to data set of *Chrysomya megacephala*, and dark purple refers to data set of *Chrysomya* (*Achoetandrus*) *ruffifacies*).

By using tSNE visualization, the AlexNet model can separate species explicitly into distinct groupings based on characteristics extracted from the model as shown in Fig. 3. All four species were separated with overlapping data of *C. megacephala* and *C. rufifacies*. It could be due to the similarity of morphological characteristics of both species^24^. This result indicated that the performance of the AlexNet model was equal to human practice.

**Figure 4:**
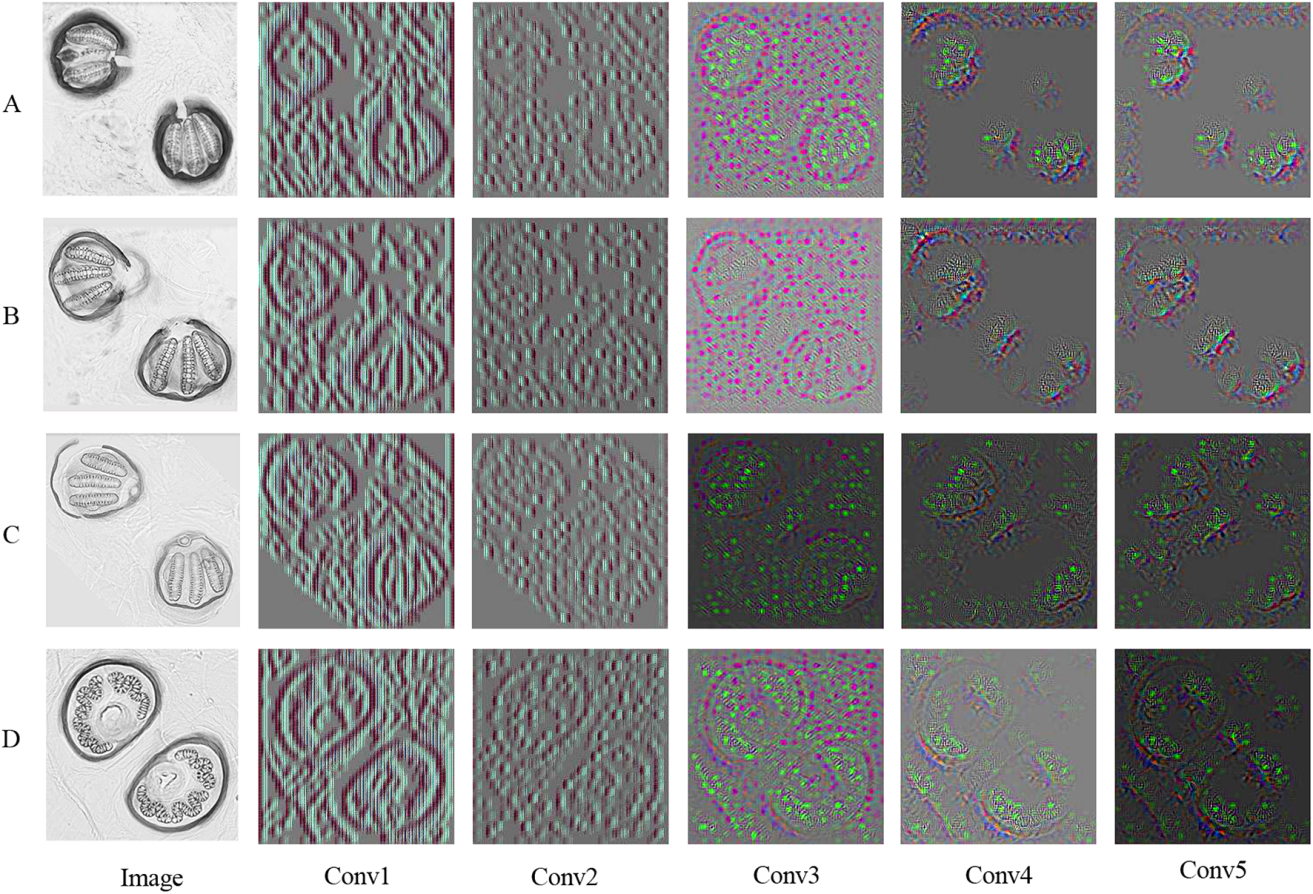
Visualization of hidden convolutional layers in AlexNet for four example images (To clearly show the patterns, we generated the color images; (**A**) *Chrysomya* (*Achoetandrus*) *ruffifacies*, (**B**) *Chrysomya megacephala*, (**C**) *Lucilia cuprina*, (**D**) *Musca domestica*).

The five hidden convolutional layers of AlexNet on four example images of posterior spiracles were visualized as shown in Fig. 4. In each layer, the posterior spiracle of each species was randomly selected for visualization. The color images were generated to clearly show the patterns. The multiple hidden layers could extract a rich hierarchy of patterns from the input image, including low-level, middle-level, and high-level patterns that can be observed. The lower layers extracted the detailed local patterns from images, such as the textures, margins, and others. The complexity and variation of the visualized patterns increased when it comes to the middle layers. The higher layers extracted specific pattern features.

**Figure 5:**
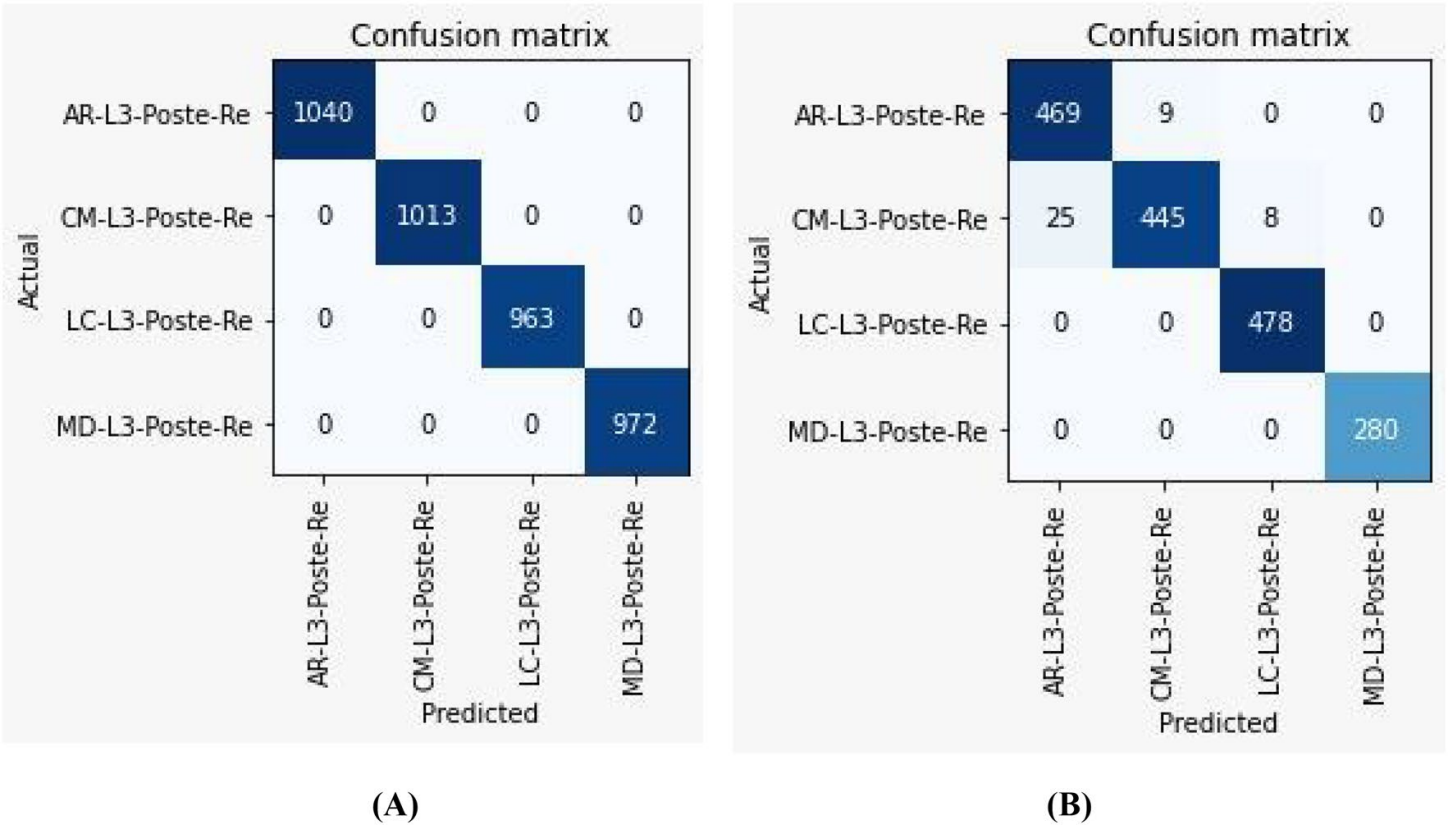
The confusion matrix achieved by the AlexNet model: (**A**) Validation (**B**) Test dataset.

**Figure 6:**
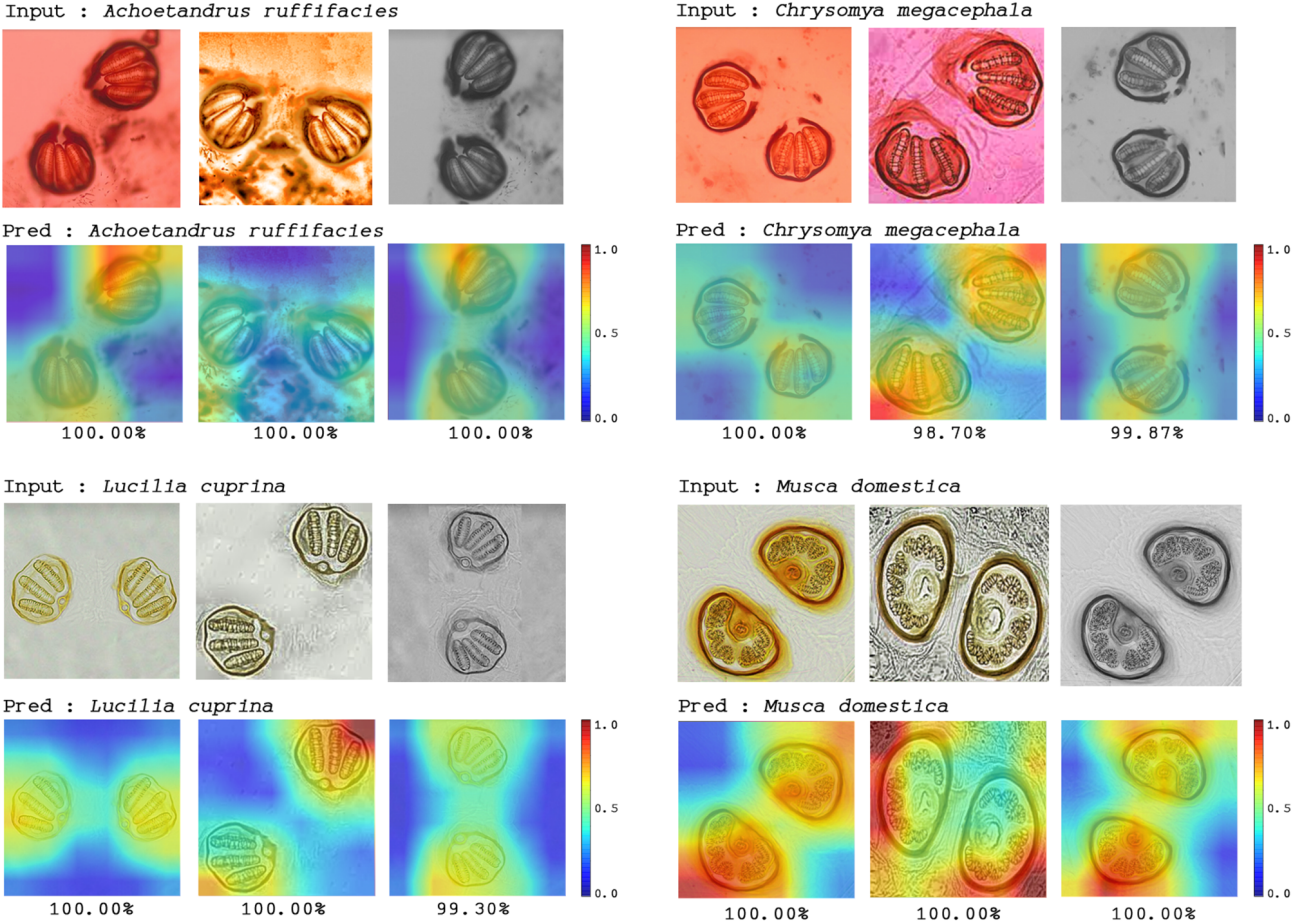
Heatmap of attention maps of AlexNet on example images showing prediction accuracy (98.70–100%) of this model for classification of each fly species in different image conditions.

The classification results (validation and test) for each image are displayed in the confusion matrices (Fig. 5) which show how predicted species (columns) correspond to the actual species (rows). The values along the diagonal indicate the number of correct predictions, whereas off-diagonal values indicate misclassifications. Interestingly, no misclassification was found after testing the model by using the test images (Fig. 5A). Therefore, the results indicated that the predictions of the AlexNet model match the taxonomic expert classification. The confusion matrix showed misclassification between *C. megacephala* and *C. rufifacies* (Fig. 5B), corresponding to the results of tSNE visualization. When the model was tested with the outsource images, the accuracy of the classification for *C. megacephala*, *C. rufifacies, L. cuprina*, and *M. domestica* was 94.94, 98.02, 98.35, 100%, respectively (Fig. 5B). The results from using the Heatmap program showed that the prediction accuracy of this model was still high (98.70–100%), depending on image conditions (Fig. 6).

**Figure 7:**
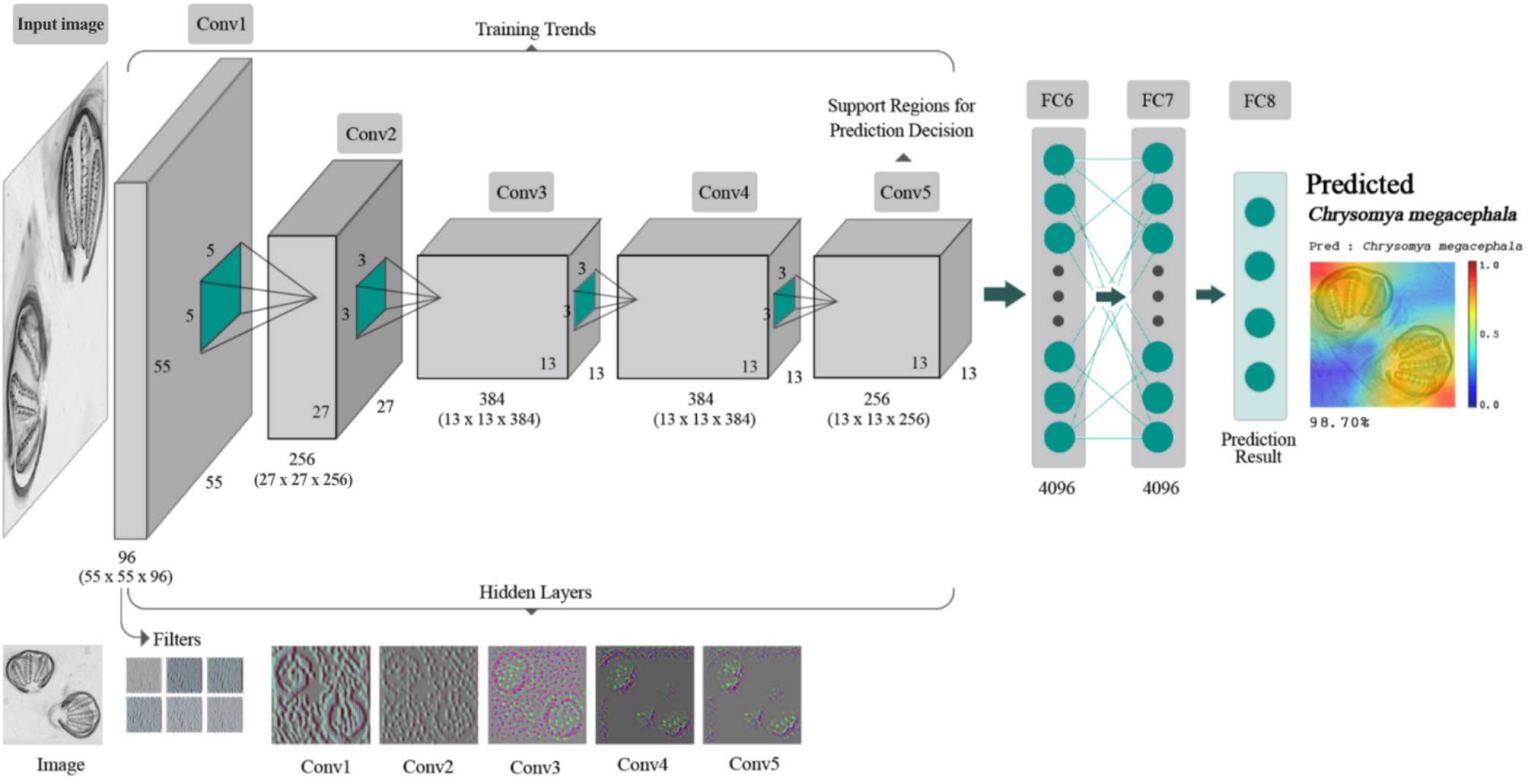
Framework of the proposed interpretation architecture for the deep learning model, AlexNet, in this study. The AlexNet model contains eight learned layers with weights (five convolutional layers and three fully connected layers), namely Conv1 as a convolutional layer that accepts an input image tensor (224 × 224 × 3) and performs Convolution to obtain the position and strength of the input image properties causing tensor output (55 × 55 × 96), Conv2 as a convolutional layer that generates tensor output (27 × 27 × 256), Conv3 as a convolutional layer that creates tensor output (13 × 13 × 384), Conv4 as a convolutional layer that creates tensor output (13 × 13 × 384), Conv5 as a convolutional layer that creates tensor output (13 × 13 × 256), FC6 as a fully connected layer (4096) which flattens tensor output from Conv5 into a single vector by weight sharing, resulting in tensor output (4096 × 1), FC7 as a fully connected layer which performs the same actions as FC6 and generates tensor output (4096 × 1), and FC8 as a fully connected layer to generates tensor output (1000 × 1) which is the prediction result and which contains 1000 neurons corresponding to the 1000 natural image classes.

The framework of the AlexNet model was demonstrated in Fig. 7. This model consists of 5 convolutional layers (Conv1, Conv2, Conv3, Conv4, and Conv5) and 3 fully connected (FC6, FC7, and FC8) layers^19^. The convolutional layers extract features, and then the fully connected layers combine the features to model the outputs. The example of characteristics of the images presented in all 5 convolutional layers were shown in Fig. 4.

Previously, CNNs have been used successfully to identify different cells or species^11–18,26,27^. This study also confirmed the efficiency of CNNs in identifying fly species.

Finally, we created a web application called “The Fly” by using our classification model for identifying species of fly maggots. The web application is available at https://thefly.ai. Users can identify species of fly maggots by uploading their images of posterior spiracles and the result with associated probability percentage will then be shown. This web application can be accessed and used on both desktop and mobile browsers. In terms of performance limitations, this web application was designed to identify only four species of fly maggots using images of posterior spiracles. This web application is the beginning step of the development of automatic species-identification for fly species in Order Diptera. More images of these four species and other species must be studied in the future. In addition, the results from this study will be applied to develop a feature as a microservice for the identification of fly maggots in a mobile application called iParasites which is currently available on AppStore and GooglePlay. We, nonetheless, wish to project that taxonomic experts are still important and critical for the development of this automatic identification by AI-based imaging system as mentioned in a previous report^11^.

## Conclusion

Currently, CNNS have been successfully used in a wide range of scientific discipline including Entomology^11–19,26,27^. This study demonstrated that all state-of-the-art CNNs used in this study including ResNet-101, Densenet161, Vgg19_bn, and AlexNet provided high efficiency in the computer vision aspect. AlexNet showed the fastest speed to process the identification model and could reliably identify species of fly maggots based on images of posterior spiracles. However, the results from this study confirmed that the AI-based imaging system will be useful in developing automatic species identification for flies and other insects. In the next step, more images of other fly species should be collected as training data sets. Moreover, the results from this study can be used in the development of identification features in mobile applications and in further species identification by using other types of images.

## Supplementary Information

Supplementary Figure S1.

## Data Availability

The dataset and source codes for this study are publicly available through the second author’s GitHub repository: https://github.com/pharinyab/Fly-PosteriorSpiracles-AlexNet.
