# Supplementary material for: Assessment of deep convolutional neural network models for species identification of forensically-important fly maggots based on images of posterior spiracles
Source: Sci Rep. 2022 Mar 19;12:4753. doi: 10.1038/s41598-022-08823-8 (PMC8934339; doi:10.1038/s41598-022-08823-8)
Supplement: Supplementary file 1 — Supplementary Figure S1. [file 41598_2022_8823_MOESM1_ESM.docx]

| 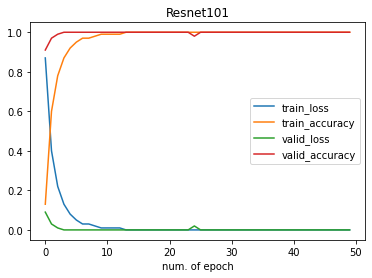 | 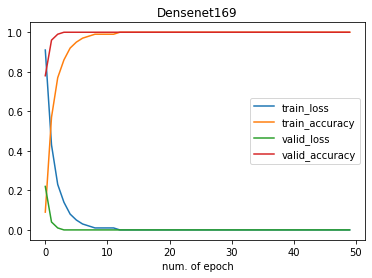 |
| --- | --- |
| 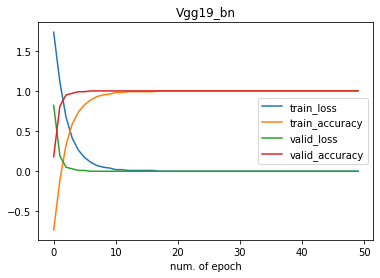 | 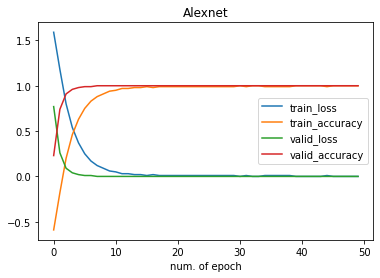 |
|  | |

**Figure S1** Validation accuracy of the four different models during the training (Supplementary data)
